# Supplementary material for: Prediction model of stock return on investment based on hybrid DNN and TabNet model
Source: PeerJ Comput Sci. 2024 Jul 25;10:e2057. doi: 10.7717/peerj-cs.2057 (PMC11639136; doi:10.7717/peerj-cs.2057)
Supplement: Supplemental Information 2 [file peerj-cs-10-2057-s002.doc]

https://figshare.com/articles/dataset/Peej_dataset/24616410
